# Towards a consensus on hearing and structure preservation in cochlear implantation surgery: results of the HEARRING international group survey

**DOI:** 10.3389/fsurg.2026.1940644

**Published:** 2026-08-31

**Authors:** Paul Van de Heyning, Shin-ichi Usami, Vedat Topsakal, Piotr H. Skarzynski, Aanand Acharya, Munir Demir Bajin, Miryam Calvino, Marco Caversaccio, Stefan Dazert, Javier Gavilán, Sara Ghiselli, Anouk Hofkens, Mohan Kameswaran, Anja Kurz, Marc Lammers, Artur Lorens, Griet Mertens, Andrea B. Overton, Narayanan Prepageran, Sasidharan Pulibalathingal, Christopher H. Raine, Ranjith Rajeswaran, Kristen Rak, Joachim Schmutzhard, Nicholas Thompson, Vincent Van Rompaey, Christiane Völter, Medhat Yousef, Mario E. Zernotti, Rudolf Hagen

**Affiliations:** 1 Department of Otorhinolaryngology, Head and Neck Surgery, Antwerp University Hospital (UZA), Edegem, Belgium; 2 Department of Translational Neurosciences, Faculty of Medicine and Health Sciences, University of Antwerp, Antwerp, Belgium; 3 Department of Hearing Implant Sciences, Shinshu University Hospital, Matsumoto, Japan; 4 Cliniques Universitaires Saint-Luc, Brussels, Belgium; 5 Institute of Neuroscience, Université Catholique de Louvain (UCL), Brussels, Belgium; 6 Department of Teleaudiology and Screening, World Hearing Center, Institute of Physiology and Pathology of Hearing, Warsaw/Kajetany, Poland; 7 Institute of Sensory Organs, Kajetany, Poland; 8 Department of Otolaryngology, Head and Neck Surgery, Fiona Stanley Fremantle Hospital Group, Perth, WA, Australia; 9 Department of Otolaryngology-Head and Neck Surgery, University Hospital – London Health Sciences Centre, London, ON, Canada; 10 Department of Otorhinolaryngology, Hospital La Paz, IdiPAZ, Madrid, Spain; 11 Biomedical Research Networking Centre on Rare Diseases (CIBERER), Institute of Health Carlos III, Madrid, Spain; 12 Department for ENT, Head and Neck Surgery, Bern University Hospital, Bern, Switzerland; 13 Department of Otorhinolaryngology, Head and Neck Surgery, St. Elisabeth Hospital, Ruhr University Bochum, Bochum, Germany; 14 University of Parma, Department of Medicine and Surgery, Parma, Italy; 15 Department of Otolaryngology, AUSL Piacenza, Piacenza, Italy; 16 Audiology Department, Madras ENT Research Foundation (MERF), Chennai, Tamil Nadu, India; 17 Department of Oto-Rhino-Laryngology, Head and Neck Surgery and the Comprehensive Hearing Center, University Clinic of Wuerzburg, Würzburg, Germany; 18 Institute of Physiology and Pathology of Hearing, World Hearing Center, Warsaw/Kajetany, Poland; 19 Department of Audiology, University of North Carolina at Chapel Hill, Chapel Hill, NC, United States; 20 Department of Otorhinolaryngology, Faculty of Medicine, Universiti Malaya, Kuala Lumpur, Malaysia; 21 Dr Manoj’s ENT Super Speciality Institute and Research Centre, Kozhikode, Kerala, India; 22 Bradford Royal Infirmary, Yorkshire Auditory Implant Service, Bradford, United Kingdom; 23 Department of Otorhinolaryngology, Head and Neck Surgery, Medical University of Innsbruck, Innsbruck, Austria; 24 Department of Otolaryngology/Head & Neck Surgery, University of North Carolina at Chapel Hill, Chapel Hill, NC, United States; 25 King Abdullah Ear Specialist Center (KAESC), College of Medicine, King Saud University Medical City, King Saud University, Riyadh, Saudi Arabia; 26 Department of Otorhinolaryngology, Sanatorio Allende de Córdoba, Córdoba, Argentina; 27 Catholic University of Córdoba and National University of Córdoba, Córdoba, Argentina

**Keywords:** cochlear Implantation, electrode array selection, Hearing preservation, sensorineural hearing loss, soft surgery techniques, Structure preservation

## Abstract

**Background:**

To evaluate current clinical practices, attitudes, and considerations regarding hearing and structure preservation (HSP) in cochlear implant surgery among international experts.

**Methods:**

A cross-sectional online survey comprising 42 structured items was distributed to 75 HEARRING members, including surgeons, audiologists, and scientists. The questionnaire covered HSP indications and candidate evaluation, electrode array selection, surgical techniques, fitting strategies, and training.

**Results:**

Thirty-one experts from 16 countries completed the survey. Most respondents (87.1%) supported HSP surgery in all patients with normal cochlear anatomy, regardless of the severity of hearing loss, primarily to preserve cochlear structures and optimize long-term outcomes. Strong consensus emerged regarding atraumatic principles, including slow electrode array insertion, flexible electrode arrays, round window access, and routine intraoperative corticosteroid use. Image-based planning was unanimously endorsed for electrode array selection, and all experts favoured longer electrode arrays even in patients with functional low-frequency hearing. Variability persisted in the adoption of genetic testing, perioperative management, intraoperative monitoring, and fitting strategies, although anatomy-based fitting was most frequently selected. Training of surgeons and audiologists was universally regarded as essential.

**Conclusions:**

This survey demonstrates a clear evolution toward a shared expert framework for HSP-oriented cochlear implantation surgery, with several principles approaching consensus. Remaining variability highlights a) areas for future research and development and b) supports the continued international collaboration toward standardized HSP practice.

## Graphical Abstract

**Figure:**
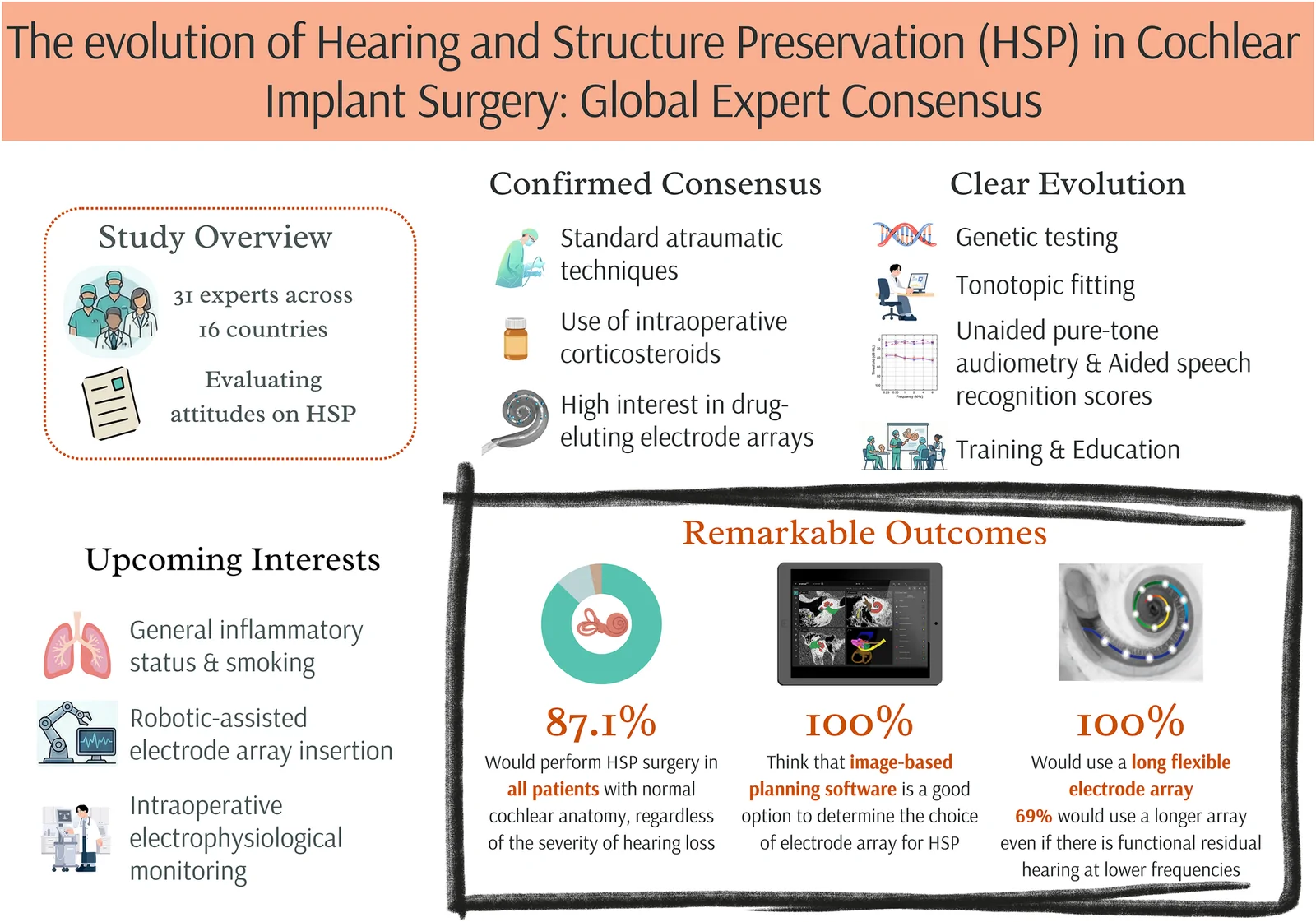

## 1 Introduction

Hearing and structure preservation (HSP) has played a central role in reshaping cochlear implantation (1). Initially, HSP focused primarily on maintaining residual low-frequency acoustic hearing to enable electro-acoustic stimulation (EAS) (2, 3). This focus was formalized through clinical guidelines defining indications, surgical principles, device considerations, and organizational requirements for adult EAS implantation (4). Over time, the principle of HSP has expanded to encompass a broader objective: minimize trauma and preserve cochlear structures for all patients undergoing cochlear implantation surgery (5, 6), making HSP a universal surgical goal rather than a niche strategy reserved for selected candidates.

Technological and methodological advances have supported this evolution. The development of flexible electrode arrays, soft surgery techniques, and pharmacological strategies such as perioperative corticosteroid use have enabled clinicians to pursue atraumatic cochlear implantation in a more systematic and reproducible manner (7–9). In parallel, expert networks such as HEARRING have facilitated international exchange of clinical experience, data sharing, and collaborative research, advancing the field.

Despite these advances, important questions remain unresolved. There is currently no up-to-date universally accepted consensus regarding which HSP principles are essential and which emerging tools and strategies should be considered standard of care (10, 11). The present international survey was therefore designed to capture current expert opinion within the HEARRING network and compare these with previous studies (12, 13). The aims were to: 1) to assess attitudes toward the universal application of HSP techniques; and 2) to identify areas of consensus and variability in surgical, pharmacological, monitoring, fitting, and training practices. By identifying current practice patterns, this study seeks to clarify the trajectory toward a shared consensus on HSP-focused cochlear implantation.

## 2 Materials and methods

### 2.1 Study design and survey content

This study was a cross-sectional survey of international cochlear implant (CI) experts. A structured questionnaire was developed by an expert panel with recognized experience in cochlear implantation, HSP surgery, and clinical research. The survey comprised 42 items and addressed 5 main domains: 1) indications for HSP and candidate evaluation, 2) electrode array selection, 3) surgical techniques, 4) fitting strategies, and 5) skills and training. Questions were formatted as multiple-choice or “select all that apply” items and included graded importance scales where appropriate. The complete questionnaire is provided in the Supplementary Materials.

### 2.2 Participants

The survey was distributed online to members of the HEARRING group from September to October 2025. Completion and return of the questionnaire were considered to indicate implied informed consent.

### 2.3 Data analysis

Responses were analysed descriptively. For each survey item, results are reported as absolute numbers and corresponding percentages to reflect the distribution of responses. For questions allowing multiple responses (i.e., “select all that apply”) or for analyses restricted to specific respondent subgroups, the denominator reflects the total number of responses or eligible respondents for that item rather than the total number of survey participants. As a result, denominators may exceed the number of participants who completed the survey. Missing responses were treated as missing values. Questionnaires were not excluded due to missing answers, and all returned questionnaires were included in the analysis. Raw survey data were exported as an excel file for initial data cleaning and structuring before being processed for analysis.

## 3 Results

### 3.1 Respondent profile

31 experts from 16 countries and 19 institutions completed the survey. For selected analyses, denominators vary due to multiple-response questions or subgroup-specific items. Respondents represented a broad geographical distribution, including Europe, Asia, North America, South America, and Australia. The sample included professionals from academic university hospitals, specialised clinical centres and implant programmes, and research-focused institutes. The majority of respondents were surgeons (58.1%, 18/31), followed by audiologists (35.5%, 11/31), and scientists (6.5%, 2/31).

### 3.2 Indications for HSP and candidate evaluation

**Figure 1:**
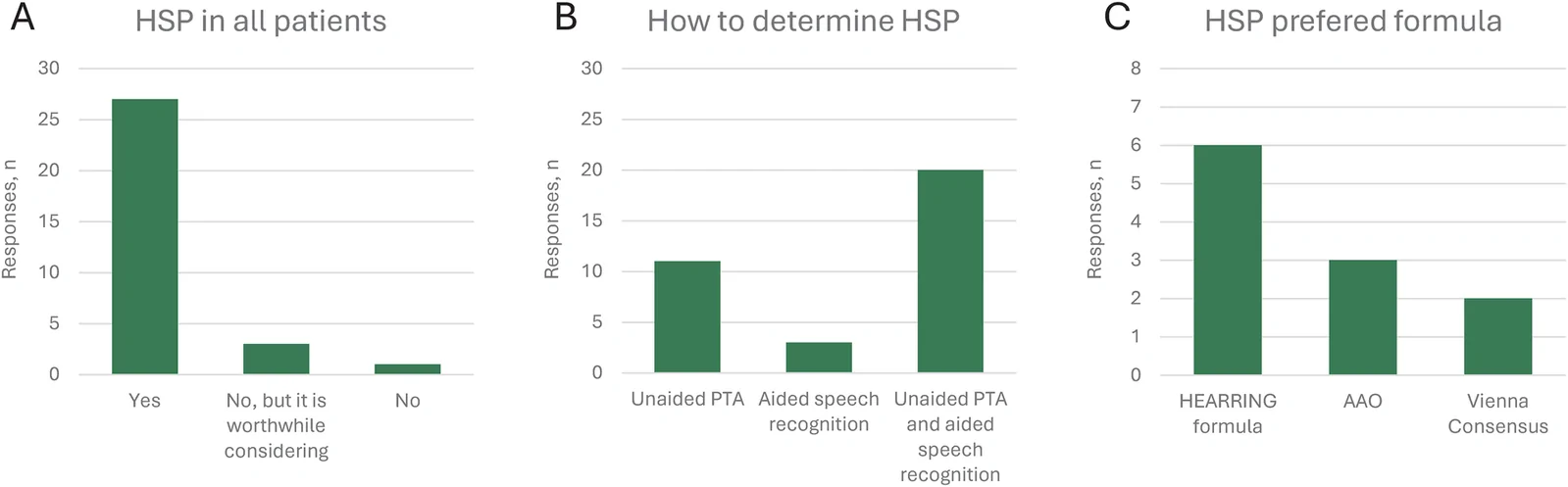
Indications for HSP and candidate evaluation. Experts’ responses to: **(A)**. ‘Would you do hearing and structure preservation (HSP) surgery in all patients with normal cochlear anatomy, including those with profound HL’, *n* = 31; **(B)**. ’Should we consider the following for determining HSP’, *n* = 34, multiple responses allowed; **(C)**. ‘Which formula would you use’, *n* = 11. The y-axis represents the number of responses **(n)**.

87.1% (27/31) would perform HSP surgery in all patients with normal cochlear anatomy, including those with profound HL, 3.2% (1/31) would not, while 9.7% (3/31) consider it worthwhile (Figure 1A). Free text responses revealed that this strong preference was driven primarily by the aim to preserve cochlear and inner ear structures (15/31), optimize outcomes (10/31), support standardization, training, and skill acquisition (10/31); and maintain eligibility for future regenerative therapies or re-implantation, and increase long-term safety (9/31).

The majority of respondents supported the combined use of unaided PTA and aided speech recognition scores for determining postoperative HSP (58.8%, 20/34). This approach was favoured over reliance on single audiometric parameters (unaided PTA: 32.4%, 11/34; aided speech recognition: 8.8%, 3/34; Figure 1B). Among those favouring a combined approach, the majority preferred the use of a formula-based method (75%, 15/20). Free text responses mentioned the need to balance audiometric measures with patient-specific factors such as age and contralateral hearing, while also noting challenges related to variability in speech testing across countries. Where unaided PTA formulas were specified, the preferred formula was the HEARRING formula (54.6%, 6/11) (14), followed by the AAO formula (27.3%, 3/11) (15), and the Vienna consensus formula (18.2%, 2/11; Figure 1C). For aided speech recognition, experts indicated that HSP could be defined using an aided monosyllabic score at 65 dB of either <30% (33.3%, 1/3), or <50% (66.7%, 2/3), reflecting differing thresholds among respondents.

Additional preoperative considerations are summarised in Table 1. With respect to additional preoperative considerations, more than half of the respondents reported that genetics should be used to obtain an indication of whether HL is stable or progressive, as this may determine if they would do a full insertion or not. Most respondents screen and treat patients for upper respiratory tract infections, while one third think that it is worthwhile considering. Fewer respondents screen patients for periodontitis. One third of the respondents strongly advised smoking cessation prior to surgery, while another third did not routinely advise it but considered it worthwhile to discuss. Vestibular function testing was performed by the majority of respondents (Table 1).

**Table 1:** Additional preoperative considerations.

Preoperative considerations	Yes	No, but it is worthwhile considering	No
Should we use genetics to define if the HL is stable or progressive?	58.6% (17/29)	n/a	41.4% (12/29)
Do you screen and treat patients for upper respiratory tract infections?	44.8% (13/29)	27.6% (8/29)	27.6% (8/29)
Do you screen and treat patients for periodontitis?	13.8% (4/29)	20.7% (6/29)	65.5% (19/29)
Do you strongly give advice not to smoke and or quit smoking 4 weeks before surgery	31% (9/29)	31% (9/29)	37.9% (11/29)
Do you test vestibular functioning?	69% (20/29)	n/a	31% (9/29)

HL: hearing loss.

### 3.3 Electrode array selection and use of image-based planning software

Electrode array selection and image-based planning software considerations are summarised in Table 2. Progressive HL (10 dB/year) was commonly reported to influence electrode array selection. Regarding electrode array length, none (0%, 0/29) would use a shorter array to match the 65 dB point, most (69%, 20/29) would use a longer array even if there is functional residual hearing at lower frequencies, and the rest (31%, 9/29) would use a longer electrode array in cases of progressive HL. OTOPLAN^TM^ (Cascination AG, Bern, Switzerland), a three-dimensional, image-based cochlear planning software that enables individualized electrode array selection and prediction of insertion depth based on patient-specific cochlear anatomy, was regarded by all respondents as a good option to determine the choice of electrode array for HSP. The majority stated that they would use the suggested array from the image-based planning software and 57.1% (12/21) would choose an angular insertion depth ≥600°. Most do not use OTOPLAN to assess the round window size, but most do use it for tonotopic fitting. Most stated that when a drug-eluting electrode array becomes available and safety requirements are met, this would be the only array that they would use for HSP and would indicate drug-eluting electrodes for all CI surgeries. Detailed responses are provided in Table 2.

**Table 2:** Electrode array selection and image planning software considerations.

Electrode array selection and image planning software considerations	Yes	No
Does progressive HL (10 dB/year) influence your choice of electrode array?	69% (20/29)	31% (9/29)
Do you think OTOPLAN is a good option to determine choice of electrode array for HSP?	100% (29/29)	0% (0/29)
Would you use the suggested electrode array from OTOPLAN?	86.2% (25/29)	13.8% (4/29)
Do you use OTOPLAN to assess the round window size?	24.1% (7/29)	75.9% (22/29)
Do you use OTOPLAN for ABF?	82.8% (24/29)	17.2% (5/29)
When a drug-eluting electrode array becomes available (and safety requirements are met), would this be the only electrode you would use for HSP?	72.4% (21/29)	27.6% (8/29)
Would you indicate drug-eluting electrode arrays for all other CI surgeries?	65.5% (19/29)	34.5% (10/29)

HL, hearing loss; HSP, hearing and structure preservation; ABF, anatomy-based fitting.

### 3.4 Surgical technique

**Figure 2:**
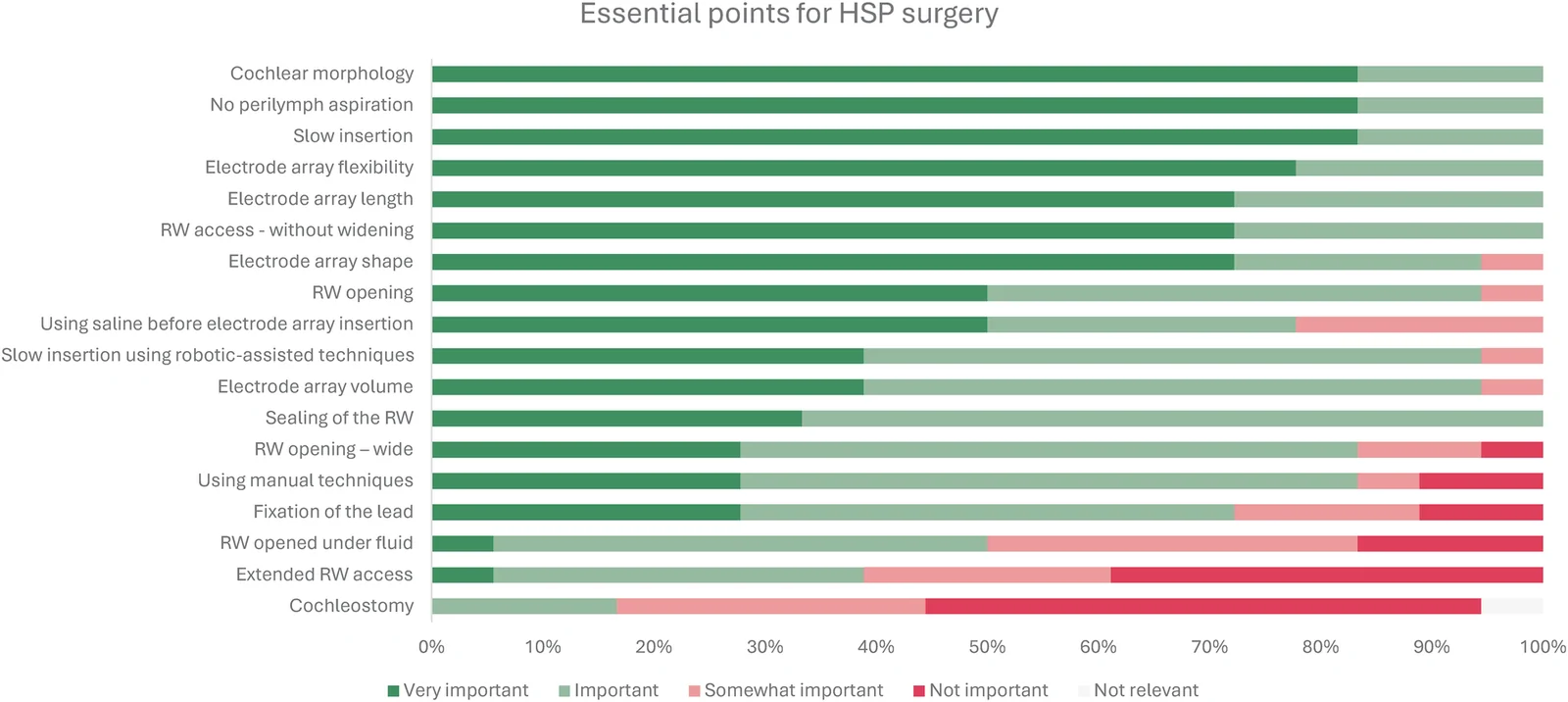
Distribution of expert ratings on the importance of surgical, anatomical, and electrode array-related factors for HSP surgery. Respondents rated each item using a five-level scale (very important, important, somewhat important, not important, and not relevant). Bars represent the percentage distribution of responses for each factor, *n* = 18.

Surgeons rated the importance of specific surgical steps, anatomical considerations, and electrode array characteristics relevant to HSP. Although responses spanned the full range of importance categories, most factors were rated as either ‘important’ or ‘very important’ (Figure 2). The aspects most frequently rated as ‘important’ or ‘very important’ were cochlear morphology; avoidance of perilymph aspiration; slow electrode array insertion; accessing the round window (RW); and array flexibility, length, and shape. RW sealing, electrode array volume, and slow insertion using robotic-assisted techniques were generally rated as ‘important’ and ‘very important’. Items such as use of saline before electrode array insertion (to clear the middle ear of bone dust and blood contamination), wide RW opening, manual insertion techniques [such as a metronome (16), or self-counting], and fixation of the lead in the posterior tympanotomy after insertion showed a broader distribution across ’somewhat important’, ‘important’, and ‘very important’. In contrast, cochleostomy, extended RW access, and RW opening under fluid received higher proportions of ’somewhat important’ and ‘not important’.

Responses regarding specific surgical practices, corticosteroid use, and intraoperative monitoring are summarized in Table 3. Consistent with Figure 2 where cochleostomy was generally considered of lower importance, with half of the respondents rating it as ‘not important’ and none rating it as ‘very important’, opinions varied regarding the optimal position and size of the cochleostomy. Half of the respondents supported centring approximately 1 mm anterior and inferior to the RW midline. A minority of respondents favoured a small cochleostomy diameter of ≤1 mm, whereas most respondents did not. In contrast, the majority supported exposure of the endosteum while keeping it intact. With respect to RW opening, a larger slit allowing perilymph outflow during insertion was more frequently selected than a smaller slit.

**Table 3:** Surgical considerations.

Surgical issues	Responses	
	Yes	No
The cochleostomy should be centred approximately 1 mm anterior and inferior to the horizontal midline of the RW	50.0% (9/18)	50.0% (9/18)
The cochleostomy should be small (≤1 mm diameter, even if the electrode tapers to 1.3 mm)	38.9% (7/18)	61.1% (11/18)
The endosteum should be exposed but kept intact	77.8% (14/18)	22.2% (4/18)
	**Large**	**Small**
RW opening size: the slit should be large or small (allowing perilymph outflow during insertion or not)	61.1% (11/18)	38.9% (7/18)

***Use of corticosteroids***	Responses	
	**Large**	**Small**
Pre-operative (day before surgery)	22.2% (4/18)	77.8% (14/18)
During surgery (at induction of anaesthesia)	94.4% (17/18)	5.6% (1/18)
During surgery (topical application)	94.4% (17/18)	5.6% (1/18)
Post-operative (over several days)	55.6% (10/18)	44.4% (8/18)
Would you use the INCAT for HSP	33.3% (6/18)	66.7% (12/18)

Intraoperative Monitoring	ECochG		ART		IFT	
Tools used for intraoperative monitoring of HSP (multiple answers allowed)	45.2% (14/31)		35.5% (11/31)		19.4% (6/31)	
	Yes	No	Yes	No	Yes	No
Do you rely on this outcome?	71.4% (10/14)	28.6% (4/14)	63.6% (7/11)	36.4% (4/11)	66.7% (4/6)	33.3% (2/6)
Does it influence your surgical handling?	100% (14/14)	0% (0/14)	45.5% (5/11)	54.5% (6/11)	66.7% (4/6)	33.3% (2/6)

RW: round window, HSP: hearing and structure preservation, ECochG: electrocochleography, ART: auditory response telemetry, IFT: impedance field telemetry, INCAT: inner ear catheter.

Perioperative corticosteroid use varied. Preoperative administration the day before surgery was uncommon, whereas intraoperative corticosteroid administration, both at induction of anaesthesia and via topical application, was widely reported. Postoperative corticosteroid use over several days was reported by approximately half of the respondents. Use of the inner ear catheter [INCAT (17)], for HSP was supported by a one third of the respondents; among those willing to use it, dexamethasone was consistently identified as the pharmacological agent of choice.

Intraoperative monitoring practices varied across respondents. Electrocochleography (ECochG) was the most frequently selected monitoring modality, followed by auditory response telemetry and impedance field telemetry. Among respondents using ECochG, most reported relying on the obtained outcome, and all indicated that it influenced their surgical handling. For auditory response telemetry (ART) and impedance field telemetry (IFT), reliance on the outcome and reported influence on surgical handling varied among respondents.

### 3.5 Fitting strategies

**Figure 3:**
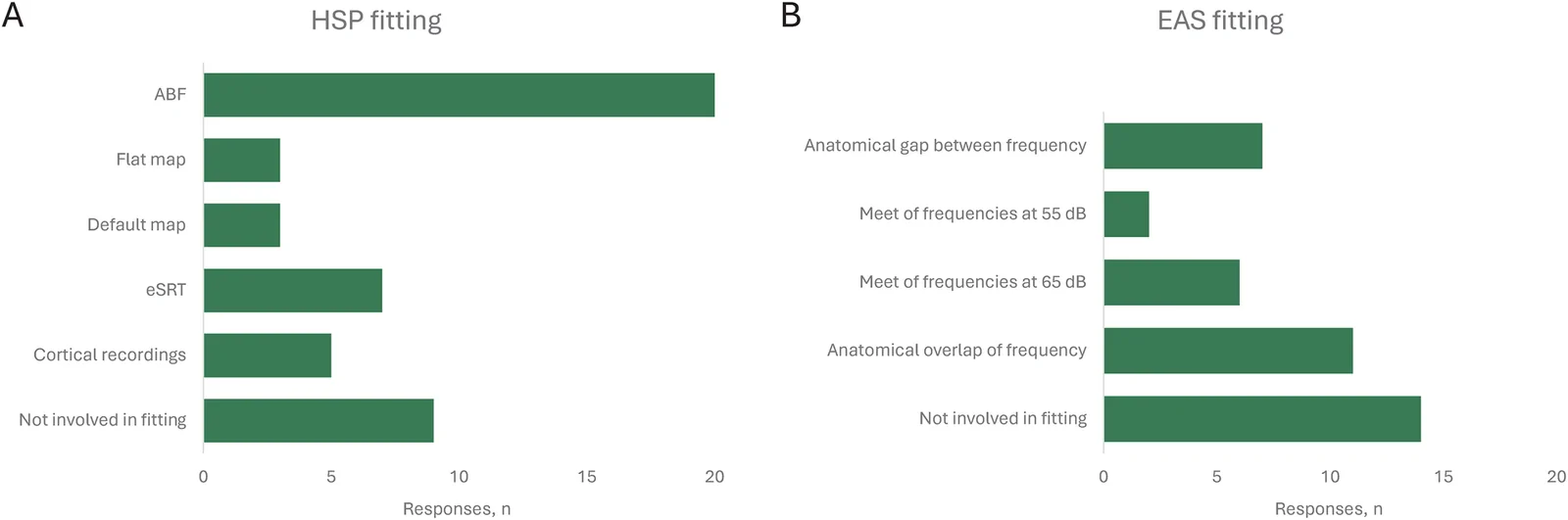
Fitting considerations. Experts’ responses to: **(A)** “Which fitting technique would you use for HSP patients?” *n* = 47, multiple responses allowed; **(B)** “Which fitting method for frequencies would you use for EAS?”, *n* = 40, multiple responses allowed. ABF: anatomy-based fitting, eSRT: electrically evoked stapedius reflex threshold, EAS: electric acoustic stimulation, HSP: hearing and structure preservation. The x-axis represents the number of respondents **(n)** for each response option.

When asked about fitting techniques used for HSP patients (multiple choices available), most respondents selected ABF (42.6%, 20/47), followed by electrically evoked stapedius reflex threshold (eSRT; 14.9%, 7/47), and cortical recordings (10.6%, 5/47; Figure 3A). When asked which fitting method they would use for frequency allocation in EAS patients, most respondents (35%, 14/40) stated that they were not involved in fitting, while among the fitting choices, anatomical overlap of frequency (27.5%, 11/40) was the preferred choice, followed by anatomical gap between frequencies (17.5%, 7/40; Figure 3B).

### 3.6 Skills/training

**Figure 4:**
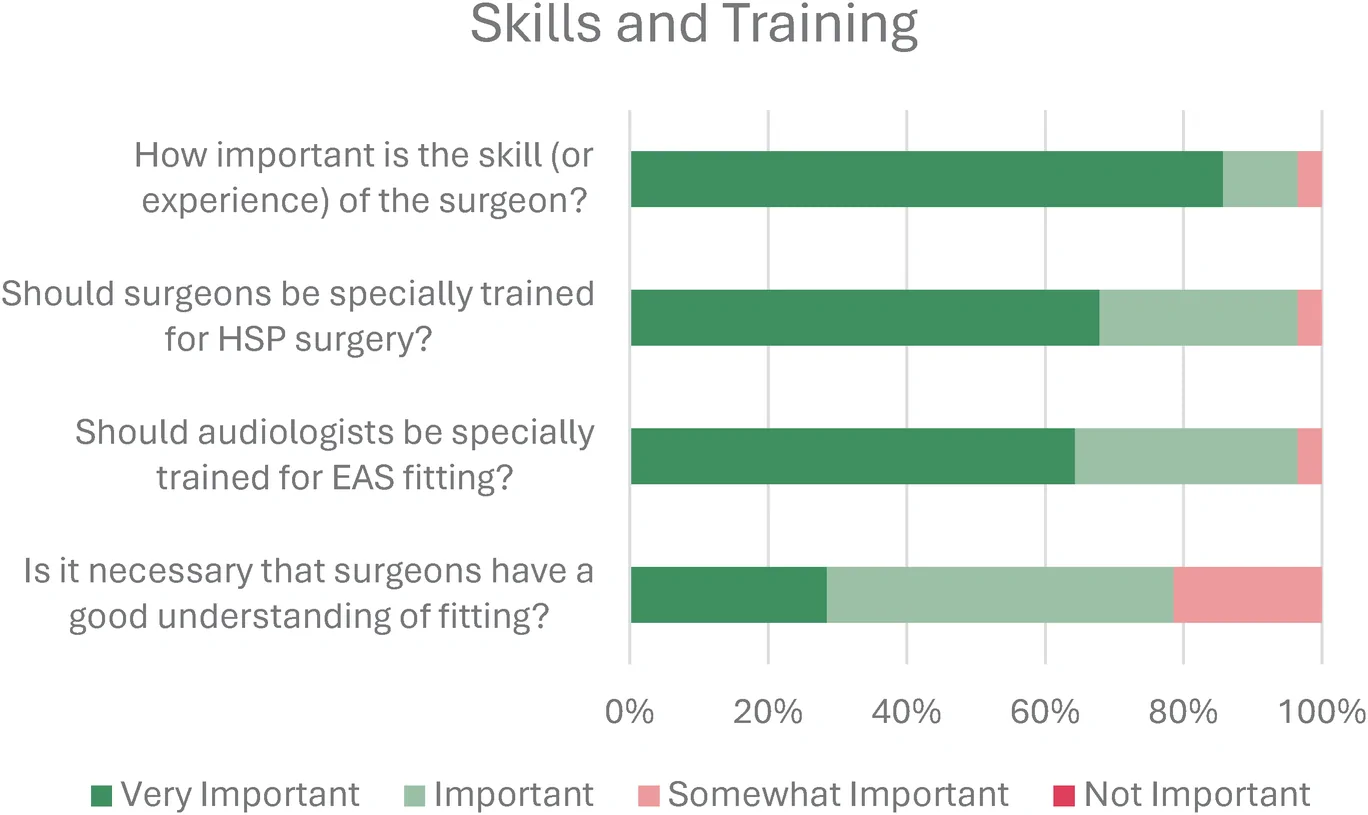
Distribution of expert ratings on the importance of skill and training. Respondents rated each item using a four-level scale (very important, important, somewhat important, not important). Bars represent the percentage distribution of responses for each factor, *n* = 28. EAS: electric acoustic stimulation, HSP: hearing and structure preservation.

Across all questions related to skills and training, no item received any ‘not important’ responses, and all domains were rated at least ’somewhat important’ by all respondents (Figure 4). Surgeon skill and experience were most frequently rated as ‘very important’ (85.7%, 24/28), followed by the need for specialized training for surgeons performing HSP surgery (67.9%, 19/28) and for audiologists conducting EAS fitting (64.3%, 18/28). Although fewer respondents rated cross-disciplinary understanding (surgeons’ understanding of fitting) as ‘very important’, the majority still considered it ‘very important’ or ‘important’ (78.6%, 22/28), indicating a broad consensus on the relevance of training and expertise.

## 4 Discussion

### 4.1 Remarkable outcomes

Survey responses revealed several findings that were surprising in a positive way, reflecting strong alignment among experts and highlighting areas of clear collective endorsement. One of the most notable outcomes was the widespread support for HSP surgery in all patients with normal cochlear anatomy, including those with profound HL. While Topsakal et al. primarily framed minimally traumatic surgery in the context of residual hearing preservation (13), the present survey demonstrates a conceptual expansion toward universal HSP, reflecting a shift from selective to routine application. This strong preference was primarily driven by the aim to preserve cochlear and inner ear structures and optimize outcomes. In addition, many surgeons advocated performing HSP in all CI cases to build routine experience, refine surgical habits, and ensure optimal technique when true hearing-preservation cases are encountered. This finding suggests that at least within this cohort, HSP is now viewed as a standard of care goal in cochlear implantation, independent of preoperative audiometric thresholds.

Another remarkable outcome was the unanimous endorsement of image-based planning software for electrode array selection in HSP. Reliance on individualized radiological measurements of the cochlea represents a substantial shift from earlier expert surveys, which reported more heterogeneous opinions (12, 13). The current 100% endorsement indicates increased trust in image-based planning software and its perceived clinical usefulness in achieving atraumatic insertion and optimized outcomes. The survey also revealed a small proportion of respondents would not invariably follow the array recommendation generated by the planning software. This finding indicates that electrode array selection remains a multifactorial clinical decision. While automated cochlear measurement software enhances usability and provides reliable anatomical information (18), factors such as anatomical variations, inner ear malformations, hearing preservation goals, and patient preferences may also influence the final choice of electrode array. These results suggest that image-based planning is regarded as a valuable decision-support tool that complements, rather than replaces, clinical judgement.

Moreover, the majority stated that they would follow the electrode array recommendation provided by the software and that they would choose an angular insertion depth of ≥600°. While insertion depth was also identified as an important factor in a previous survey, no specific angular threshold had been defined (13). This finding is noteworthy given the substantial variability in cochlear size observed across different populations (19). Recent anatomical evidence based on synchrotron radiation phase-contrast imaging suggests that an angular insertion depth exceeding 600° may be necessary to achieve complete spiral ganglion coverage (20). Furthermore, clinical evidence supports the potential functional benefits of greater cochlear coverage, with Canfarotta et al. demonstrating superior speech-in-noise performance in recipients implanted with longer (31.5 mm) compared with medium-length (24 mm) lateral wall electrode arrays (21). These findings provide a possible explanation for why many HEARRING experts favoured insertion depths of ≥600° in the current survey.

Additionally, all experts in this survey favoured longer electrode arrays and many reported routine use of long electrode arrays, even in candidates with functional low-frequency hearing. This approach appears to be driven by the high prevalence of progressive hearing loss among candidates. Experts aim to preserve residual hearing in the short term while anticipating the future need for deeper cochlear coverage (22). Contemporary evidence demonstrates that medium and long flexible lateral wall electrode arrays achieve similarly high short- and medium-term hearing preservation rates (23). Therefore, implantation of a long array during the primary surgery should be considered to reduce the likelihood of future reimplantation. Moreover, multiple studies have shown that complete cochlear coverage and deeper insertion depths are associated with better speech understanding outcomes, both in the short and long term (21, 24–26). Emerging data show that also in EAS, the use of long electrode arrays resulted in successful hearing preservation in all cases while providing superior cochlear coverage compared with shorter electrodes (27). Collectively, these findings support the expert consensus that selecting a long electrode array to achieve complete cochlear coverage (i.e., a coverage of more than 1 ½ turns, corresponding to 600° angular insertion depth) represents a dual-purpose strategy that balances immediate HSP with long-term auditory rehabilitation.

### 4.2 Confirmed consensus

Several elements reached a level of agreement consistent with a confirmed consensus, indicating that these practices are considered standardized for HSP-focused cochlear implantation (12, 13). Across respondents, there was a strong agreement on avoiding perilymph aspiration, performing slow electrode array insertion using flexible electrode arrays, and preferentially inserting through the round window rather than alternative access routes. These principles align with longstanding atraumatic concepts and were consistently rated as important or very important.

Corticosteroid use also emerged as an area of strong agreement consistent with a confirmed consensus (13). The vast majority of respondents reported intraoperative systemic and topical corticosteroid administration. Moreover, a large number of the respondents showed interest in using a drug-eluting electrode array (28, 29) not only for HSP, but also for all other CI surgeries when one is available and the safety requirements are met. The high level of agreement in the use of corticosteroids reflects growing confidence in the safety and potential protective effects of corticosteroids during cochlear implantation.

### 4.3 Towards a consensus

Beyond areas of established agreement, the results presented in Figure 2 demonstrate a clear movement toward consensus across a broader range of surgical and technical factors. Importantly, none of the evaluated items were considered irrelevant or unimportant by the majority of respondents, in contrast to earlier surveys (12, 13). The absence of ‘negative’ ratings reflects a more unified understanding of the multifactorial nature of HSP.

Fixation of the electrode lead after insertion was also rated as highly important (30, 31), supported by emerging evidence that intracochlear pressure changes—occurring with both short and long electrodes—may influence HSP outcomes (32). Fixation of the electrode lead would also be important for flexible electrode arrays, due to the risk of electrode array slippage after implantation (33).

Electrode array volume emerged as another factor of growing importance. While previously underappreciated, electrode array volume is now increasingly recognized as a potential contributor to intracochlear pressure and trauma (34, 35). This shift indicates that attention is moving beyond electrode array length alone toward a more comprehensive evaluation of array–cochlea interaction.

In contrast, interest in cochleostomy-based approaches appears to be declining. Indeed, histopathologic examinations after electrode array insertion via cochleostomy showed substantial intracochlear fibrosis, ossification, and hydrops (36, 37). When cochleostomy had to be used, respondents were divided on the optimal position but tended to favour a larger (>1 mm) diameter. In contemporary practice, cochleostomy is often used due to an inability to adequately access the round window, rather than by preference. This reinforces the view that the round window is the dominant access route in contemporary HSP practice.

### 4.4 Indications of increasing alignment towards a new consensus

The survey suggests a shift toward greater alignment in several domains extending beyond surgical technique. One such domain is genetic testing. While this was not addressed in earlier surveys (12, 13), the current results show that more than half of respondents believe genetic information should be used to determine whether hearing loss is stable or progressive (38). Importantly, this reflects an increased willingness to incorporate genetics into clinical decision-making and indicates that it is not universally implemented at present (39).

Tonotopic fitting, which was the most frequently selected fitting strategy, also reflects this evolution. Historically, tonotopic fitting required postoperative CT imaging, however recent advances have enabled its implementation using standard X-ray imaging (40–42). Given that tonotopic fitting is already widely used, simplification of the required imaging is expected to further increase its clinical adoption.

Audiometric indication criteria show a preference toward a combined approach using unaided pure-tone audiometry and aided speech recognition scores. While low-frequency PTA remains central to candidacy selection, many experts reported using the HEARRING formula (14) to quantify and compare pre- and postoperative hearing preservation outcomes. This finding is consistent with the growing number of peer-reviewed studies that have adopted the HEARRING formula for outcome evaluation (17, 43–46).

Finally, training and education emerged as a universally endorsed priority, even more so than previously reported (13). No respondent rated training-related items as ‘not important’, indicating a unanimous recognition of training as a prerequisite for successful HSP implementation. Surgeon skill and experience were rated as ‘important’ or ‘very important’ by nearly all respondents, reflecting the central role of surgical expertise in achieving atraumatic insertion and minimizing intracochlear damage. Similarly, there was strong agreement that surgeons should be specifically trained for HSP surgery, with most respondents considering such training ‘very important’, showing that HSP requires dedicated competencies beyond conventional cochlear implantation techniques.

From an audiological perspective, respondents agreed that it is ‘important’ or ‘very important’ for audiologists to receive specific training for EAS fitting, reflecting the close interdependence between surgical preservation and postoperative programming strategies. In addition, a substantial proportion of respondents considered it ‘important’ or ‘very important’ that surgeons have a good understanding of fitting principles, emphasizing the need for cross-disciplinary knowledge and close collaboration between surgeons and audiologists. Taken together, these results indicate a clear consensus that HSP is not solely technique-dependent but relies on specialized training, accumulated experience, and integrated multidisciplinary expertise.

### 4.5 Emerging areas of interest

Several topics were identified as areas of emerging and sustained interest. One such area is the evaluation of general inflammatory status, including smoking behaviour (47) and infectious foci such as upper respiratory tract infections. Although not uniformly implemented, existing evidence from middle-ear surgery indicates that continued smoking is associated with substantially worse outcomes (48). This suggests that systemic and local inflammatory factors may meaningfully influence HSP results in CI surgery and these factors are likely to gain increasing importance in future clinical evaluations.

Robotic-assisted electrode array insertion also represents an emerging field of interest. Early clinical reports suggest reduced trauma and improved hearing preservation outcomes with robotic insertion (49–54). As this technology continues to evolve, robotic-assisted insertion is expected to become increasingly common in clinical practice, especially in HSP surgery and in combination with perioperative electro-physiologic assessments (55).

Finally, intraoperative electrophysiological monitoring, and particularly electrocochleography, was not universally used, but those who employed it reported high confidence (56) in its value and consistently modified their surgical behaviour based on the obtained signals (57). Similar to robotic-assisted electrode array insertion, interest is likely to increase as evidence accumulates.

Beyond the topics addressed in this survey, an area of growing interest is scala tympani volume and its relationship to electrode array design. This concept builds on recent work demonstrating interindividual variability in scala tympani volume (35). Despite a correlation between scala tympani volume and cochlear duct length, scala tympani volume was a better predictor of HSP than cochlear duct length in that study, suggesting that individual cochlear morphology may have a greater impact on HSP outcomes than insertion depth when using current lateral wall electrode arrays and atraumatic surgical techniques. In a separate retrospective analysis, Räth et al. further supported the relevance of scala tympani volume by showing that cochlear volume had the tendency to be larger in patients with hearing preservation than in those with hearing loss (58).

### 4.6 Limitations

The data of this survey largely reflect perspectives within the HEARRING network, an international collaborative group of hearing implant centres focused on sharing experience and working toward consensus in clinical practice. As a result, responses reflect only the opinions of the survey participants and may be influenced by familiarity with concepts and technologies commonly associated with the HEARRING network. Moreover, as this study was designed as an opinion survey, respondents were not asked to justify their individual choices, and the precise rationale underlying some of their preferences can only be inferred. In addition, the extent to which these findings apply to CI systems from different manufacturers is unclear, as certain features discussed are not uniformly available or emphasized across brands. Notwithstanding these limitations, a key strength is the multidisciplinary composition of the respondents, including surgeons, audiologists, and scientists. This diversity provides a comprehensive view across clinical and research domains.

## 5 Conclusion

Our findings confirm and extend the conclusions of previous surveys, indicating increasing alignment from early consensus on atraumatic surgical principles toward a more comprehensive, technology-integrated, and widely adopted HSP framework. High levels of agreement were observed on key elements such as round window access, slow insertion using flexible electrode arrays, routine intraoperative corticosteroid administration, and image-based electrode array length planning. The comparison further indicates how concepts that were still evolving or debated in 2020 have, in many cases, consolidated into common practice. The consistent emphasis on specialized training and multidisciplinary collaboration indicates that successful HSP depends not only on technological advances but also on expertise and coordinated care, supporting continued international efforts toward formal consensus guidelines.

## Data Availability

The raw data supporting the conclusions of this article will be made available by the authors, without undue reservation.
