# Supplementary material for: Towards a consensus on hearing and structure preservation in cochlear implantation surgery: results of the HEARRING international group survey
Source: Front Surg. 2026 Aug 31;13:1940644. doi: 10.3389/fsurg.2026.1940644 (PMC13572638; doi:10.3389/fsurg.2026.1940644)
Supplement: Supplementary file 1 [file Table1.docx]

Supplementary Material

# 1 HSP Questionnaire

1. Are you a: (Surgeon/ Audiologist/ Clinical Engineer/ Scientist)
2. Would you do HSP surgery in all patients (also in case of profound HL) with normal cochlear anatomy you operate on? (Yes/ No/ No, but it is worthwhile considering)
3. Why?

**Indications for HSP/ Defining Hearing Preservation**

4. Should we use genetics to define if the hearing loss is stable or progressive, which may determine if you do a full insertion or not? (Yes/ No)
5. Should we consider the following for determining HSP: (Unaided PTA/ Aided speech recognition/ Unaided PTA and aided speech recognition)
   a. If unaided PTA was selected: Which formula would you use? (HEARRING Adunka/ Vienna Consensus)
   b. If aided speech recognition was selected: What aided monosyllable score presented at 65 dB would show HP? Less than: (30%/ 40%/ 50%/ 60%)
   c. If unaided PTA and aided speech recognition was selected: How would you combine unaided PTA and aided speech recognition? (With a formula (to be determined)/ Another idea? Please state)

**Candidate evaluation**

6. Do you screen and treat patients for upper respiratory tract infections? (Yes/ No/ No, but it is worthwhile considering)
7. Do you screen and treat patients for periodontitis? (Yes/ No/ No, but it is worthwhile considering)
8. Do you strongly give advice not to smoke and/or quit smoking 4 weeks before surgery? (Yes/ No/ No, but it is worthwhile considering)
9. Do you test vestibular functioning? (Yes/ No)

**Electrode array choice**

10. Does progressive hearing loss (10 dB/ year HL) influence your choice of electrode? (Yes/ No)
11. Would you use: (A longer electrode in case of progressive hearing loss/ A shorter electrode to match the 65 dB point/ A longer electrode even if there is functional residual hearing at lower frequencies?)
12. Do you think OTOPLAN is a good option to determine choice of electrode for HSP? (Yes/ No)
13. If OTOPLAN is selected: Would you use the suggested electrode? (Yes/ No)
14. If OTOPLAN is selected: With which angular insertion depth? (Free text)
15. If OTOPLAN is selected: Do you use OTOPLAN to assess the round window size? (Yes/ No)
16. If OTOPLAN is selected: Do you use OTOPLAN for Anatomy Based Fitting? (Yes/ No)
17. When a drug-eluting electrode becomes available (and safety requirements are met), would this be the only electrode you would use for HSP? (Yes/ No)
18. Would you indicate drug eluting electrodes for all other CI surgeries? (Yes/ No)

**Surgical techniques (only for surgeons)**

19. Which are the essential points for HSP surgery. Click all that are relevant for you. Clicking ‘Very Important’ means that this point would be a compulsory action for HSP (very important/ important/ somewhat important/ not important/ not relevant):
    a. Cochlear morphology (CDL using preop radiological imaging to measure the cochlear duct length).
    b. Electrode length.
    c. Electrode flexibility.
    d. Electrode volume.
    e. Electrode shape (straight or precurved).
    f. Using saline before electrode insertion to clear the middle ear of bone dust and blood contamination.
    g. Round window access (this method does not require widening of RW; however, it may include removing bony overhang).
    h. Extended round window access (this method requires widening the entrance of the RW).
    i. Cochleostomy (this method requires opening the cochlear at a different place compared to the RW).
    j. Should be centred approximately 1mm anterior and inferior to the horizontal midline of the round window.
    k. No perilymph aspiration
    l. RW opening: do you favour large or small slit (allowing perilymph outflow during insertion or not)
    m. RW opened under fluid
    n. RW opening – wide
    o. Slow insertion
    p. Using manual techniques (metronome, self-counting)
    q. Slow insertion using robotic assisted insertion such as OTODRIVE
    r. Sealing of the RW
    s. Fixation of the lead in the posterior tympanotomy after insertion
20. The cochleostomy should be centered approximately 1 mm anterior and inferior to the horizontal midline of the RW (Yes/ No)
21. The cochleostomy should be small, no more than 1mm in diameter (even if the electrode tapers to 1.3 mm) (Yes/ No)
22. Expose the endosteum but keep it intact (Yes/ No)
23. RW opening: do you favor large or small slit (allowing perilymph outflow during insertion or not) (Large slit/ Small slit)

**Use of corticosteroids**

24. Do you use corticosteroids? Pre-operative, the day before (Yes/ No)
25. Do you use corticosteroids? During surgery, at induction of anaesthesia (Yes/ No)
26. Do you use corticosteroids? During surgery, topically (Yes/ No)
27. Do you use corticosteroids? Post-operatively over several days (Yes/No)
28. Would you use the INCAT for HSP? (Yes/ No)
    a. If yes was selected: What drug and dosage would you use?
29. What tools would you use during surgery to monitor HSP? Tick all that apply (ECochG/ ART/ IFT)
    a. If ECochG was selected: Do you rely on this outcome? (Yes/ No)
    b. If ECochG was selected: Does it influence your surgical handling? (Yes/ No)
    c. If ART was selected: Do you rely on this outcome? (Yes/ No)
    d. If ART was selected: Does it influence your surgical handling? (Yes/ No)
    e. If IFT was selected: Do you rely on this outcome? (Yes/ No)
    f. If IFT was selected: Does it influence your surgical handling? (Yes/ No)

**Fitting**

30. Which fitting technique would you use for HSP patients? Click all that apply. (Anatomy based fitting (ABF)/ Flat map/ Default map/ eSRT/ Cortical recordings/ Not involved in fitting)
31. Which fitting method for frequencies would you use for EAS? Click all that apply. (Anatomical Gap between frequency/ Meet of frequencies at 55 dB/ Meet of frequencies at 65 dB/ Anatomical Overlap of frequency)

**Skills**

32. How important is the skill (or experience) of the surgeon? (very important/ important/ somewhat important/ not important)
33. Should surgeons be specially trained for HSP surgery? (very important/ important/ somewhat important/ not important)
34. Should audiologists be specially trained for EAS fitting? (very important/ important/ somewhat important/ not important)
35. Is it necessary that surgeons have a good understanding of fitting? (very important/ important/ somewhat important/ not important)
